# Distinct Phenotypic and Genomic Signatures Underlie Contrasting Pathogenic Potential of *Staphylococcus epidermidis* Clonal Lineages

**DOI:** 10.3389/fmicb.2019.01971

**Published:** 2019-08-27

**Authors:** Diana Espadinha, Rita G. Sobral, Catarina Inês Mendes, Guillaume Méric, Samuel K. Sheppard, João A. Carriço, Hermínia de Lencastre, Maria Miragaia

**Affiliations:** ^1^Laboratory of Bacterial Evolution and Molecular Epidemiology, Instituto de Tecnologia Química e Biológica António Xavier, Universidade Nova de Lisboa, Oeiras, Portugal; ^2^Laboratory of Molecular Genetics, Instituto de Tecnologia Química e Biológica António Xavier, Universidade Nova de Lisboa, Oeiras, Portugal; ^3^Laboratory of Molecular Microbiology of Bacterial Pathogens, UCIBIO/REQUIMTE, Faculdade de Ciências e Tecnologia, Universidade Nova de Lisboa, Costa de Caparica, Portugal; ^4^Molecular Microbiology and Infection Unit, Instituto de Medicina Molecular, Faculdade de Medicina de Lisboa, Universidade de Lisboa, Lisbon, Portugal; ^5^The Milner Centre for Evolution, University of Bath, Bath, United Kingdom; ^6^MRC CLIMB Consortium, Bath, United Kingdom; ^7^Laboratory of Microbiology and Infectious Diseases, The Rockefeller University, New York, NY, United States

**Keywords:** *S. epidermidis*, pan genome, GWAS, clonal lineages, pathogen, commensal

## Abstract

**Background:** *Staphylococcus epidermidis* is a common skin commensal that has emerged as a pathogen in hospitals, mainly related to medical devices-associated infections. Noteworthy, infection rates by *S. epidermidis* have the tendency to rise steeply in next decades together with medical devices use and immunocompromized population growth. *Staphylococcus epidermidis* population structure includes two major clonal lineages (A/C and B) that present contrasting pathogenic potentials. To address this distinction and explore the basis of increased pathogenicity of A/C lineage, we performed a detailed comparative analysis using phylogenetic and integrated pangenome-wide-association study (panGWAS) approaches and compared the lineages’s phenotypes in *in vitro* conditions mimicking carriage and infection.

**Results:** Each *S. epidermidis* lineage had distinct phenotypic signatures in skin and infection conditions and differed in genomic content. Combination of phenotypic and genotypic data revealed that both lineages were well adapted to skin environmental cues. However, they appear to occupy different skin niches, perform distinct biological functions in the skin and use different mechanisms to complete the same function: lineage B strains showed evidence of specialization to survival in microaerobic and lipid rich environment, characteristic of hair follicle and sebaceous glands; lineage A/C strains showed evidence for adaption to diverse osmotic and pH conditions, potentially allowing them to occupy a broader and more superficial skin niche. In infection conditions, A/C strains had an advantage, having the potential to bind blood-associated host matrix proteins, form biofilms at blood pH, resist antibiotics and macrophage acidity and to produce proteases. These features were observed to be rare in the lineage B strains. PanGWAS analysis produced a catalog of putative *S. epidermidis* virulence factors and identified an epidemiological molecular marker for the more pathogenic lineage.

**Conclusion:** The prevalence of A/C lineage in infection is probably related to a higher metabolic and genomic versatility that allows rapid adaptation during transition from a commensal to a pathogenic lifestyle. The putative virulence and phenotypic factors associated to A/C lineage constitute a reliable framework for future studies on *S. epidermidis* pathogenesis and the finding of an epidemiological marker for the more pathogenic lineage is an asset for the management of *S. epidermidis* infections.

## Background

*Staphylococcus epidermidis* is one of the most abundant commensal bacteria of healthy human skin and mucosa. This organism has emerged in recent decades as an important opportunistic pathogen, being the main cause of nosocomial infections associated to indwelling medical devices such as peripheral or central intravenous catheters (CVCs) (Otto, 2009). Infections by *S. epidermidis* usually occur due to a breach in the skin barrier resulting from the insertion of the medical devices, allowing *S. epidermidis* to penetrate the host tissues.

The progression to infection after skin penetration depends on the ability of *S. epidermidis* to rapidly change from a commensal to a pathogenic state. As skin commensals, *S. epidermidis* survive and grow under nutrient limitation, at a low temperature (<37°C) and pH (∼4.5–6.4) (Schmid-Wendtner and Korting, 2006) and at diverse osmotic pressures resulting from the production/evaporation of sweat and fluctuations in environmental humidity (Wilson, 2005). Furthermore, they have to cope with cell desquamation, and oxidative stress resulting from UV exposure (Kammeyer and Luiten, 2015). Once in the bloodstream, upon skin barrier breach, the environmental landscape changes dramatically and *S. epidermidis* suddenly face a nutrient-rich and alkaline environment with a higher temperature, pro-inflammatory molecules and reactive oxygen species (ROS) generated by immune cells (Akira et al., 2006; Weiss and Schaible, 2015) and eventual antibiotic pressure (Ciofu et al., 2017). However, the factors of *S. epidermidis* that contribute for the transition from health to disease state are not completely understood.

One of the factors thought to be crucial for transition from skin to blood is the formation of biofilms on the surface of medical devices, which can be composed of a mesh of proteins, exopolysaccharides and extracellular DNA (Qin et al., 2007; Otto, 2009). These biofilms can confer protection against the host immune system (Vuong et al., 2004) and resistance to antibiotics (Farber et al., 1990; Khardori et al., 1995; Singh et al., 2010), making infections extremely difficult to treat (Otto, 2009). Other mechanisms that have been shown to be important for *S. epidermidis* pathogenicity include the ability to evade human innate immunity, namely through processes involved in resistance to antimicrobial peptides (AMP) (Cheung et al., 2010). Besides the *ica* operon, which is directly involved in biofilm formation (Heilmann et al., 1996) and the insertion sequence IS256, shown to modulate biofilm formation and antibiotic resistance (Kozitskaya et al., 2004), other virulence factors have been proposed. These include the primary attachment to host extracellular matrices and intercellular aggregation, toxins, proteases and lipases, all suggested to be implicated in invasive potential (Otto, 2012).

Whole genome sequence analysis (Conlan et al., 2012; Meric et al., 2015) has shown that the *S. epidermidis* population was composed of two main phylogenetic clusters: lineage A/C, containing most of the isolates from colonization and infection and lineage B, comprising mainly colonization isolates. In spite of their clinical relevance, the factors associated to the success of A/C strains, both as colonizers and pathogens, are not fully understood.

Here we hypothesize that the success of A/C strains as colonizers and as pathogens is associated with an increased ability to adapt to environmental constraints imposed by both colonization and infection states. To address this, we analyzed the genomes of representative isolates from A/C and B clusters and characterized their phenotypic traits in conditions that mimic the colonization and infection scenarios.

## Results and Discussion

### *S. epidermidis* Population Structure Is Composed of Two Clusters With Different Pathogenic Potential

In previous studies we have characterized a collection of 1714 *S. epidermidis* from different isolation dates, geographic and clinical origins (Miragaia et al., 2007; Rolo et al., 2012). Based on multilocus sequence typing (MLST) data we selected a collection of 83 isolates that represent the diversity of strains in terms of genetic backgrounds (71 sequence types, between 1996 and 2001). To understand how isolates of different origins were related, we generated a pan genome based on the annotated genomes from 82 *S. epidermidis* strains under study (one genome was excluded from the genomic analysis due to possible contamination, see Materials and Methods) and compared the genomic content of the strains according to their isolation source. The pangenome obtained had a total size of 5.0 Mbp with 31.3% of GC content, encoding a total of 6682 genes. From these, approximately one third (*n* = 1653) comprised the core genome (present in all strains in this study), with the remainder (5029) comprising the accessory genome. Gene accumulation curves that plot pan genome size as a function of the number of genomes sequenced, suggest an open pan genome that is characteristic of populations that undergo frequent horizontal gene transfer (HGT) (Meric et al., 2015) (Supplementary Figure S1).

**FIGURE 1:**
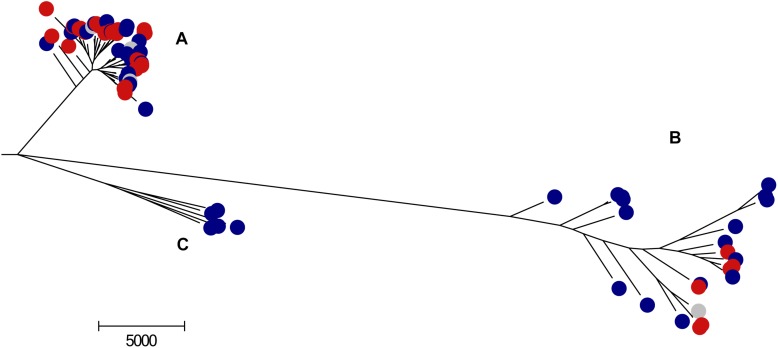
Maximum-likelihood tree of the 82 *S. epidermidis* strains. The tree was generated by RaxML (Stamatakis et al., 2012) embedded in Gubbins (Croucher et al., 2015), based on the core gene alignment with removal of the recombining regions. In blue are depicted the colonization isolates, in red the infection isolates and in gray are the isolates for which no epidemiological information was available. **(A–C)** Correspond to *S. epidermidis* clonal clusters, according to Meric et al. (2015).

Phylogenetic reconstruction, based on the Maximum-likelihood (ML) tree derived from the core genome corrected for recombining regions, confirmed that the *S. epidermidis* population is divided into three clusters (Figure 1). Consistent with previous studies (Meric et al., 2015), Clusters A (66%) and C (6%) were joined in a single cluster and were analyzed together in further analysis. The remainder of the isolates belonged to cluster B (28%). The A/C cluster contained mainly nosocomial isolates (*n* = 49/59, 83%) whereas the B cluster contained a higher proportion of isolates from healthy people in the community (*n* = 13/23, 56%) (*p* = 0.0007), confirming previous findings by Conlan et al. (2012) for a much smaller and geographically delimited isolate collection. A/C cluster isolates almost equally originated from infection (*n* = 28/59, 47%) and colonization (*n* = 31/59, 53%) sources, while B cluster isolates were mostly from colonization (17/23, 70%) (*p* = 0.127) even though this different distribution did not reach statistical significance. These results suggest that although both A/C and B strains are carried asymptomatically, A/C strains are, apparently, more implicated in infection events. This observation is corroborated by multiple studies wherein *S. epidermidis* from infection were in their majority from A/C cluster (which comprises clonal complex 2 as defined by MLST) (Miragaia et al., 2007; Widerstrom et al., 2009; Gordon et al., 2012; Hellmark et al., 2013; Cherifi et al., 2014).

### Evidence for the Clonal Dissemination of A/C Cluster Isolates

An important characteristic of some opportunistic pathogens is the ability to disseminate in different hosts and environments. This can lead to broad distribution of closely related strains. The mean number of SNP differences between the core genomes of A/C and B clusters was 39889, which correspond to 1.6% of the average genome size of *S. epidermidis*. Within each cluster, the average number of SNPs was similar for strains of cluster B (average number of SNP differences = 16786, ranging between 31 and 36796) and for strains of cluster A/C (average number of SNP differences = 17696 nucleotides, ranging between 1 and 37617). The lower number of differences (<100 SNPs) were found between 11 pairs of A/C isolates and only one pair in cluster B, suggesting that A/C isolates are more clonal than B isolates. Furthermore, these pairs included isolates from different countries, isolated either from the hospital or the community, demonstrating the dissemination of this cluster through these settings.

In contrast with other known nosocomial pathogens, including *S. aureus* (Ruimy et al., 2009; Grundmann et al., 2010), we found no evidence of geographic clustering in *S. epidermidis*, since each branch in the ML tree, corresponding either to A/C or B clusters (considering a maximum of 50 SNPs difference), contained isolates from multiple countries (see Supplementary Table S1). Furthermore, there were eight pairs of strains from different countries that, with one exception, belonged to the A/C cluster that diverged in less than 100 SNPs and that were isolated within the same year. This was the case for strains from Denmark and Portugal (68 SNPs; DEN161, 966N), Denmark and Iceland (60 SNPs; DEN116, ICE122) and Argentina and Hungary (17 SNPs; AGT24, HUR105), among others (see Supplementary Table S1). These examples suggest extensive and rapid geographic dissemination of strains belonging to the A/C cluster, likely promoted by skin-to-skin contact between travelers. This is consistent with previous studies that hypothesized broad geographic dissemination of this species (Miragaia et al., 2002; Widerstrom et al., 2006).

### Evidence for Ecological Isolation and Distinct Biological Functions of A/C and B Clusters

The success of a bacterial clone may be influenced by its capacity to adapt to different environments and this is frequently accomplished by the acquisition of exogenous genetic elements (Uhlemann et al., 2012; Walther et al., 2018), which will compose the accessory genome. To assess differences in gene content of the two *S. epidermidis* clusters, we analyzed and quantified accessory genome variation. The matrix for presence/absence of all the genes in the genomes is represented in Supplementary Table S2. The majority of A/C and B pan genomes was composed of accessory genes (A/C: 74%, 3723/5029; B: 67%, 3381/5029). Among this accessory genome, 1648 genes were exclusive to A/C cluster and these genes varied in frequency from 93% to 2% and 1306 genes were exclusive to cluster B and ranged from 100 to 4%, suggesting high genome plasticity and cluster-specific functions. The number of accessory genes shared between strains of the same cluster (3723 in A/C and 3381 genes in B) is 1.8–1.6 higher than the number of genes shared between the two clusters (2075 genes). This difference could be due to the existence of a barrier to genetic transfer between them. However, in contrast to *S. aureus*, this apparently low frequency of genetic transfer is not evidently associated with the presence of Restriction/Modification (R-M) systems (Waldron and Lindsay, 2006; Corvaglia et al., 2010) and/or the clustered regularly interspaced short palindromic repeats (CRISPR) (Marraffini and Sontheimer, 2008). There was no clear association of a *hsdR*/*hsdM* system to any of the clusters and *cas* and *csm* genes were equally distributed among strains of the two clusters [A/C: 12% and B: 17% (*p* > 0.05)] (Supplementary Table S2). An alternative explanation for the lower level of genetic exchange between the clusters, in this apparently recombinogenic species (Miragaia et al., 2007; Meric et al., 2015), might be the existence of tropism of each of these lineages to specific skin niches (Grice et al., 2009; Oh et al., 2014) that could have lead to some ecological isolation of the two genetic clusters. Actually, skin microbiota composition was previously shown to vary according to the environmental characteristics of the skin ecological niche sampled (Costello et al., 2009; Grice et al., 2009). To test the hypothesis of ecological isolation we have examined the seven MLST genes, which constitute a good representation of the core genes, either in terms of genome distribution and genetic diversity (Gomes et al., 2005; Miragaia et al., 2007). Data showed an apparent allelic segregation, where *aro*E gene presented no common alleles between the clusters and the remaining genes (*arc*C, *gtr*, *mut*S, *pyr*R, *tpi*, *yqi*L), comprising between 11 and 20 alleles, had a maximum of two alleles shared by the two clusters.

Furthermore, we looked for genes that were significantly associated to each cluster using a pan genome wide-association approach, that scores genes for associations with specific epidemiological features. In particular, 166 genes were identified to have a strong positive association (Benjamini–Hochberg *p* < 0.05) with cluster A/C (see Supplementary Table S3) and 244 genes with a strong association with cluster B (see Supplementary Table S4). Tables 1, 2 include the genes among these, which have functions that were either present exclusively in one of the clusters or that were present in both but in significantly different frequencies. Strains of the A/C cluster were enriched for genes involved in processes such as biofilm formation and adhesion to host matrix proteins, proteolysis, resistance to antibiotics and adaptation to low pH. Conversely, strains from cluster B were enriched for genes involved in the detoxification of formate and formaldehyde, oxidative stress response, host interaction through type VII secretion system, lipid metabolism and cell wall biosynthesis. This is consistent with *S. epidermidis* clusters A/C and B performing distinct biological functions and possibly occupying different skin niches.

**TABLE 1:** Genes positively associated to cluster A/C (Benjamini–Hochberg *p* < 0.05 and OR > 1).

**Putative functions**	**Gene annotation**	**Functional annotation**	**Frequency in cluster A/C %**	**Frequency in cluster B %**
Hydrolysis	group_1452	Putative hydrolase	92	35
Adhesion and Biofilm	*sdr*F	Serine-aspartate repeat-containing protein F	68	9
	*sdr*I	Putative surface protein	63	9
	*ica*A^∗∗^	Poly-beta-1,6-*N*-acetyl-D-glucosamine synthase	37	4
	*ica*B^∗∗^	Poly-beta-1,6-*N*-acetyl-D-glucosamine *N*-deacetylase	37	4
	*ica*C^∗∗^	Putative poly-beta-1,6-*N*-acetyl-D-glucosamine export protein	37	4
	*ica*D^∗∗^	Poly-beta-1,6-*N*-acetyl-D-glucosamine synthesis	37	4
	*ica*R^∗∗^	Biofilm operon icaADBC HTH-type negative transcriptional regulator	37	4
Nickel and Cobalt uptake	*nmt*R^∗^	HTH-type transcriptional regulator NmtR	37	0
Arginine deiminase	*arc*D^∗∗^	Arginine/ornithine antiporter	55	4
Penicillin resistance	*bla*I	Penicillinase repressor	68	17
	*bla*Z	Beta-lactamase	60	22
Copper resistance	*mco^∗∗^*	Multicopper oxidase mco	68	30
Proteolysis	*spl*F^∗∗^	Serine protease SplF	78	35
Carbohydrate metabolism	*gno^∗∗^*	Gluconate 5-dehydrogenase	62	22
	*ywq*N^∗∗^	Putative NAD(P)H-dependent FMN-containing oxidoreductase YwqN	37	4
	*ydj*H^∗^	Putative sugar kinase YdjH	37	4
	group_1735	Hexose-6-phosphate:phosphate antiporter uhpT	34	0
Thiamine uptake	*thi*Q	Thiamine import ATP-binding protein ThiQ	80	30
Cysteine biosynthesis	*cys*L	HTH-type transcriptional regulator CysL	88	43

*^∗^Only one gene/allele for that function and function is exclusive in cluster; ^∗∗^Only one gene/allele for that function and is enriched in the cluster but the function is not exclusive in cluster.*

**TABLE 2:** Genes positively associated to cluster B (Benjamini–Hochberg *p* < 0.05 and OR > 1).

**Putative functions**	**Gene annotation**	**Functional annotation**	**Frequency in cluster B %**	**Frequency in cluster A/C %**
Formate detoxification	group_862 group_863	Putative formate dehydrogenase Putative formate dehydrogenase	91 22	0 0
Formaldehyde assimilation/detoxification	*hxl*R_1	HTH-type transcriptional activator HxlR	22	0
Copper resistance	*cue*R^∗^	HTH-type transcriptional regulator	22	0
Oxidative stress response	group_3122 (*crt*O)^∗∗^(?)	Glycosyl-4,4′-diaponeurosporenoate acyltransferase	22	0
	*crt*P^∗∗^	Diapolycopene oxygenase	22	0
	*crt*Q^∗∗^	4,4′-diaponeurosporenoate glycosyltransferase	22	0
	*crt*M^∗∗^	Dehydrosqualene synthase	22	0
	*crt*N^∗∗^	Dehydrosqualene desaturase	22	0
Host-interaction and Virulence	*esa*A^∗^	ESAT-6 secretion accessory factor	65	0
	*esa*B^∗^	ESAT-6 secretion accessory factor	65	0
	*ess*C^∗∗^	ESAT-6 secretion machinery protein	65	0
	*esx*A^∗^	ESAT-6 secretion system extracellular protein A	65	0
	*ess*B^∗∗^ group_2414^∗∗^	ESAT-6 secretion machinery protein ESAT-6 secretion machinery protein	39 30	0 0
	*yez*G_1 *yez*G_2 *yez*G_3 *yez*G_5	Putative antitoxin Putative antitoxin Putative antitoxin Putative antitoxin	52 48 43 26	0 0 0 2
Carbohydrate metabolism	*tre*A^∗^	Trehalose-6-phosphate hydrolase	26	0
	*mtl*R^∗^	Transcriptional regulator MtlR	22	0
	*mtl*A^∗^	PTS system mannitol-specific EIICB component	22	0
Lipid metabolism	group_3907^∗^	NADP-dependent 7-alpha-hydroxysteroid dehydrogenase	48	0
Benzoate degradation	*lig*I^∗^	2-pyrone-4,6-dicarbaxylate hydrolase	26	0
Transport systems	group_1444	ABC transporter ATP-binding protein NatA	43	0
Cell wall biosynthesis	*dac*A	D-alanyl-D-alanine carboxypeptidase DacA	39	2
	*yod*J^∗∗∗^	Putative carboxypeptidase YodJ	35	2
Transposable elements	*bin3*_3	Tn552 DNA-invertase	43	0
	*int-tn*	Tn916 transposase Int-Tn	87	34

*^∗^Only one gene/allele for that function and function is exclusive in cluster; ^∗∗^More than one gene/allele for that function but function is exclusive in cluster; ^∗∗∗^Only one gene/allele for that function and is enriched in the cluster but the function is not exclusive in cluster.*

### *S. epidermidis* of A/C and B Clusters Have Adapted to Skin Environment Conditions Using Different Strategies

Skin is the first barrier of the human body against external aggressions and contains molecules that derive from the metabolism of skin cells and our microbiota, but also from the environment (Wilson, 2005). On the skin, *S. epidermidis* have to deal with nutrient limitation, harsh and variable environmental conditions and mechanic stresses. Perspiration is one of the central contributors for the composition of skin milieu and a major physiological function that assists in thermoregulation, skin surface hydration and immune defense. Perspiration is composed mainly of water, but contains several other metabolites that contribute for its multiple functions, such as antimicrobial peptides (AMP), immunoglobulins, natural moisturizing factors (lactate, urea, electrolytes, amino acids), vitamins, metals and salts (Wilson, 2005).

To understand if the *S. epidermidis* clusters differ in their ability to adapt to skin we have compared their ability to grow and produce biofilm in conditions that mimic the skin environment (acidic pH and increased salt concentrations). Moreover, we looked in more detail for the genes that were associated by the pan genomic wide-association approach to each lineage and that could provide an advantage in the skin environmental conditions.

#### Growth and Biofilm Formation at Acidic pH

**FIGURE 2:**
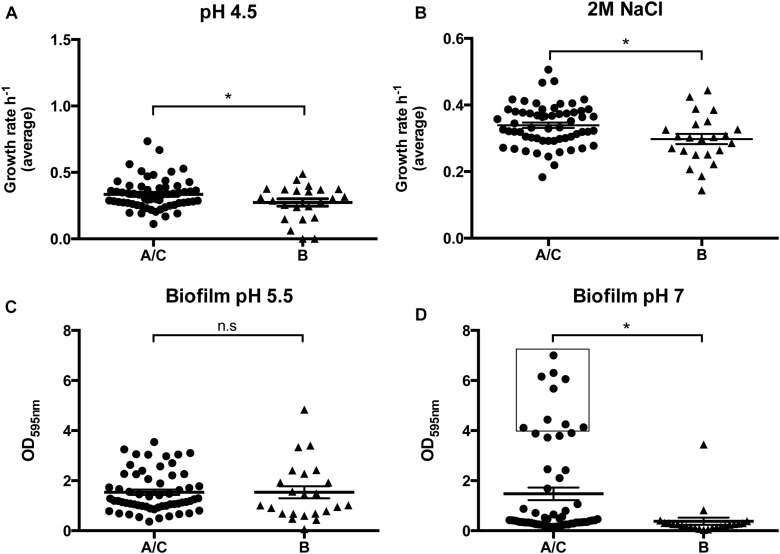
Performance of cluster A/C and B strains in the growth and biofilm assays. **(A)** Average growth rates for A/C and B strains grown in liquid medium at pH 4.5; **(B)** Average growth rates for A/C and B strains grown in liquid medium with 2M NaCl; **(C)** Average biofilm formation capacity for A/C and B strains at pH 5.5; **(D)** Average biofilm formation capacity for A/C and B strains at pH 7.

*Staphylococcus epidermidis* belonging to A/C cluster appear to have a distinctive ability to adapt to the two acidic pH values tested (pH 4.5 and pH 5.5), as strains from the A/C cluster reached, in average, higher cell densities in stationary phase (∼12% higher in both cases) and presented either a greater (pH4.5: μ_*A/C*_ = 0.335; μ_*B*_ = 0.274, *p* = 0.043) or similar average growth rate (pH 5.5: μ_*A/C*_ = 0.626; μ_*B*_ = 0.594, *p* = 0.273) when compared to strains from the B cluster (see Figure 2A and Table 3). Additionally, strains belonging to the A/C cluster were associated to a gene involved in the arginine deiminase metabolism (*arcD*, an arginine/ornithine antiporter) (see Table 1) usually carried by the arginine catabolic mobile element (ACME). Although a genomic copy of *arc*D gene with a similar function is known to be ubiquitous in the *S. epidermidis* genome, this extra copy together with the remaining *arc* operon within ACME was previously suggested to be important for pH homeostasis in *S. epidermidis* and to contribute to additional tolerance to low-pH in community-associated *Staphylococcus aureus* USA300 clone (Thurlow et al., 2013; Lindgren et al., 2014). Other functions associated to A/C cluster strains that can eventually contribute for the observed increased growth rate is the flavin-containing oxidoreductase (YwqN), the YdjH sugar kinase; and the hexose-6-phosphate:phosphate antiporter (UhpT) (Park et al., 2015), which lead either to the production of reducing power (NADH) for the biosynthesis of major cell components or to the rescue of alternative sugar sources.

**TABLE 3:** Performance of cluster A/C and cluster B strains in skin and blood conditions.

**Conditions tested**	**Cluster A/C**	**Cluster B**	***p*-value**
**Skin**			
Growth at pH 5.5			
Growth rate (h^–1^)	0.626	0.594	0.273
Lag phase (min)	234	212	–
Final OD (nm)	1.369	1.231	–
Growth at 2M NaCl			
Growth rate (h^–1^)	0.339	0.298	0.011
Lag phase (min)	361	404	–
Final OD (nm)	1.231	1.08	–
Biofilm at pH 5.5 (OD_595 *nm*_)	1.538	1.536	0.994
**Blood**			
Growth at pH 7.4			
Growth rate (h^–1^)	0.788	0.728	0.122
Lag phase (min)	191	212	–
Final OD (nm)	1.456	1.389	–
Biofilm at pH 7 (OD_595 *nm*_)	1.475	0.377	0.0003
Adhesion to collagen (OD_595 *nm*_)	0.185	0.175	0.671
**Skin and Blood**			
Growth at pH 4.5			
Growth rate (h^–1^)	0.335	0.274	0.043
Lag phase (min)	234	276	–
Final OD (nm)	0.591	0.528	–
Growth at 0.15 M NaCl			
Growth rate (h^–1^)	0.701	0.670	0.411
Lag phase (min)	212	212	–
Final OD (nm)	1.476	1.470	–
Antioxidant capacity (nmol/μL)	10.20	10.79	0.428
Cell wall charge (OD_410 *nm*_)	20.10	27.09	0.084
Proteolysis			
High (no isolates)	29	4	
Medium (no isolates)	21	14	0.032
Low (no isolates)	10	5	
Penicillin resistance			
no. resistant isolates (%)	52 (87%)	12 (48%)	
no. susceptible isolates (%)	8 (13%)	11 (9%)	0.002
Oxacillin resistance			
no. resistant isolates (%)	37 (62%)	10 (43%)	
nr. susceptible isolates (%)	23 (38%)	13 (57%)	0.147
Gentamicin resistance			
no. resistant isolates (%)	21 (35%)	2 (9%)	
no. susceptible isolates (%)	39 (65%)	21 (91%)	0.026

The two groups could not be distinguished by their ability to produce biofilm at pH 5.5 (OD_*A/C*_ = 1.538 *vs.* OD_*B*_ = 1.536, *p* = 0.994), since almost all strains, irrespective of their genetic background, produced biofilm in this condition, suggesting that biofilm is important for the growth of this commensal at the surface of the skin (see Table 3 and Figure 2C).

#### Growth in Osmotic Stress Conditions

The better capacity of the strains from the A/C cluster to withstand perspiration, osmotic stress and desiccation conditions frequently imposed at the skin surface was apparent from their capacity to grow at sodium chloride concentrations characteristic of the skin (0.15M NaCL) (Schittek et al., 2001) and in high salinity stress (2M NaCl) (Price-Whelan et al., 2013). Whereas at a physiological concentration (0.15M NaCl), strains from cluster A/C and B had equivalent average growth rates (μ_*A/C*_ = 0.701 *vs.* μ_*B*_ = 0.670, *p* = 0.411) (see Table 3), at osmotic stress conditions (2M NaCl), the medium growth rate of A/C cluster strains was significantly higher (μ_*A/C*_ = 0.339 *vs.* μ_*B*_ = 0.298, *p* = 0.011; see Figure 2B). Furthermore, they showed a much shorter lag phase (A/C: 361 min *vs*. B: 404 min), and reached higher final ODs (14% higher) (see Table 3). The results suggest that, in contrast to *S. epidermidis* strains of B cluster, strain from A/C cluster are well adapted to a range of salinity concentrations suggesting improved survival in a broader range of niches.

We found that different genes indirectly involved on resistance to osmotic stress were specifically associated to either cluster A/C or B. This is the case of genes which were associated to A/C cluster that are involved in the production of polysaccharide intercellular adhesion (PIA) (*icaADBCR* operon), which through the production of biofilm induce protection against fast osmotic changes by decreasing the rate of water loss and rehydration (Roberson and Firestone, 1992; de Goffau et al., 2009) (see Table 1). Furthermore, genes associated to B cluster that could induce osmoprotection include those involved in: peptidoglycan cross-linking (*dacA* - a *pbp4* homolog, *yodJ*) that provides extra rigidity to the bacterial cell wall (Wyke et al., 1981; Loskill et al., 2014); and the uptake of mannitol (*mtlA, mtlR*), a known osmolyte (Kets et al., 1996; Zahid et al., 2015). The absence of *mtlD*, responsible for mannitol degradation, suggests that strains of B cluster use mannitol mainly as an osmolyte rather than as a carbon or energy source (da Costa et al., 1998).

Although strains of both clusters carry genes that are associated to resistance to osmotic stress, overall, the processes used by strains of the A/C cluster appear to be more efficient than those of strains from the B cluster, as illustrated by its higher growth rate *in vitro*.

#### Genetic Traits Associated to Evasion of Skin Innate Immunity

Skin is continuously exposed to commensal microorganisms and challenged to defend the human body from invasion by pathogens. To deal with this extensive microbial exposure, skin tissues produce antimicrobial peptides (AMPs) and free fatty acids that target the bacterial cell wall or cell membrane structures (Kenny et al., 2009; Gallo and Hooper, 2012). To survive, bacteria have developed multiple strategies, including the production of carotenoid pigments that protect from free fatty acids and the inactivation of AMPs by proteolysis.

We found that strains belonging to the B cluster more commonly contained the operon involved in staphyloxanthin biosynthesis (*crtOPQMN*) that was described to confer resistance to free fatty acids produced by skin cells (Chamberlain et al., 1991). The production of the carotenoid was confirmed phenotypically for the group of five strains carrying this operon through the observation of production of a yellow color by colonies grown on agar growth medium. Other pigments had been found to be synthesized by *S. epidermidis* (Ogo, 1985), but this is the first time that staphyloxanthin operon is described in this species. We also found a serine-protease (SplF) positively associated to strains from cluster A/C (Table 1). Staphylococcal proteases have already been implicated in the mechanism of defense against the human innate immune system, as some of these proteases have the ability to cleave and inactivate human AMPs, namely dermcidin (Lai et al., 2007) and LL-37 (Sieprawska-Lupa et al., 2004) that are produced by epithelial cells and although this could be the case also for *S. epidermidis* SplF, this has yet to be proven. Altogether, the results suggest that both *S. epidermidis* of A/C and B cluster are equipped to evade the host immune system and that could be a major advantage for survival in both skin and blood.

#### Interaction With the Host and Pathogens

Commensal bacteria are in constant interaction with the surrounding environment and the host. Secretion systems are central elements in these interactions but have been investigated mostly from the perspective of pathogen-host interactions. Recent data suggest that these systems may play a role in mutualistic relationships between bacteria and the host (Silver et al., 2007) or in mediation of bacterial interactions (Basler et al., 2013). We found that *S. epidermidis* strains belonging to B cluster were associated to genes related to type VII secretion system (T7SS) (Lai et al., 2007). In particular, all the conserved T7SS structural genes (*esaA, esaB, essA, essB, essC*) were co-located in the chromosome. Moreover, there was considerable strain-to strain variation in the number and type of secreted toxin/antitoxin-homologs downstream this operon (see Table 2).

T7SS were previously described in *S. epidermidis* and were proposed to secrete an inhibitory toxin against *Propionibacterium acnes* (Christensen et al., 2016), another common commensal of the skin. Yet, in *S. aureus*, the functions of T7SS are broader, involving not only intraspecies competition (Cao et al., 2016), but also persistence and pathogenicity (Burts et al., 2008; Kneuper et al., 2014; Korea et al., 2014). The identification of a T7SS in *S. epidermidis* belonging to B cluster is consistent with the hypothesis that *S. epidermidis* of cluster B and *Propionibacterium* may share a similar niche in the skin.

### *S. epidermidis* Genetic Content Reflects Adaptation to Anthropogenic Activities

Previous studies have shown that human skin composition is also defined by our daily routines (Bouslimani et al., 2015), including activities that imply frequent contact with the skin, like hygiene habits and the application of skin care products and clothing. Moreover, sweat has been considered for long as a cleanser, allowing the excretion of toxic substances that could have been absorbed due to acute or chronic exposure to contaminated environment (air, water, food) or to consumed products. We found that *S. epidermidis* of either A/C or B cluster were associated to six loci related to the uptake, detoxification or regulation of metabolites (gluconate, trehalose, copper, and formaldehyde) that according to the Human Metabolome Database^1^ (Wishart et al., 2018) are present in the skin tissues and that are known to be simultaneously present in the composition of food, pharmaceutical products, detergents/disinfectants and/or cosmetics (see Tables 1, 2).

#### Degradation of Sugar and Sugar Derivatives

Genes involved in the degradation of sugars or sugar derivatives, namely trehalose (*treA*) and gluconate (*gno*), were associated with B and A/C clusters respectively, suggesting that strains from the two clusters use these alternative sugars as carbon and energy sources. The use of gluconate as a carbon source and its central role in bacterial cell division and colonization was previously described for other bacteria like *Streptococcus suis* (Shi et al., 2014) and *Escherichia. coli* (Sweeney et al., 1996).

Trehalose is not commonly used by *S. epidermidis* as a carbon source. Actually, the absence of trehalose fermentation has been considered a signature of *S. epidermidis* used to distinguish it from other bacterial species (Kloos and Schleifer, 1975). A possible origin for trehalose found in the skin, besides food and cosmetics (Ohtake and Wang, 2011), are bacteria of the genus *Propionibacterium* (also part of skin natural flora), which are described to be capable of synthesizing this sugar (Piwowarek et al., 2018). The results suggest once more that *Propionibacterium* and *S. epidermidis* of B cluster might share the same niche, possibly hair follicles and sebaceous regions wherein *Propionibacterium* are enriched (Scharschmidt and Fischbach, 2013) and the trehalose produced by *Propionibacterium* can be used by *S. epidermidis* as a carbon and energy source.

Having the capacity to metabolize gluconate and trehalose, either from anthropogenic or microbial origin, can be highly advantageous for *S. epidermidis* in an environment of nutrient limitation like the skin.

#### Resistance and Modulation of Copper

*Staphylococcus epidermidis* strains belonging to A/C cluster and B cluster were associated to genes involved in copper regulation, although using different mechanisms. While strains from the A/C cluster were associated to multicopper oxidase (*mco*), an enzyme involved in the oxidation of Cu^+^ to Cu^2+^ (Sitthisak et al., 2005), B cluster strains were associated to *cue*R, a MerR-family metalloregulatory transcriptional activator that senses intracellular Cu^+^ and upregulates *cop*A and *cue*O, two genes involved in copper efflux and oxidation in response to increasing copper concentrations (Chaturvedi and Henderson, 2014). Copper is utilized by bacteria for several essential metabolic processes, but is also critical for the maintenance of a healthy skin, namely for the synthesis and stabilization of extracellular matrix skin proteins and angiogenesis (Raju et al., 1982; Philips et al., 2012). Although an important enzyme cofactor and signaling molecule, the intracellular levels of free copper can be damaging for bacteria, leading to ROS-related oxidative stress derived from its reduction and oxidation cycles, reason by which it is frequently added to cosmetics as a biocide. Copper oxidation performed by systems like Mco and Cue probably help *S. epidermidis* to tightly regulate and maintain copper homeostasis in the skin (Festa and Thiele, 2012; Chaturvedi and Henderson, 2014).

#### Detoxification of Formaldehyde

*Staphylococcus epidermidis* belonging to B cluster were additionally associated with a gene (*hxlR*) involved in detoxification of formaldehyde, a metabolite that can be highly deleterious to human cells. The formaldehyde-responsive transcriptional factor *hxlR* is part of the formaldehyde detoxification/assimilation via a ribulose monophosphate (RuMP)-dependent pathway, through which formaldehyde is converted into fructose-6 phosphate and subsequently incorporated into the usual carbon metabolism pathway (Chen et al., 2016). Formaldehyde, is ubiquitously found in nature and is an important metabolite of both bacteria and human cells (Burgos-Barragan et al., 2017), but is also one of the most synthesized compounds as the result of anthropogenic activities. Toxicity of formaldehyde to animals, humans and bacteria (Tang et al., 2009; Chen et al., 2016) is due to the fast reaction of formaldehyde with free thiol (−SH) and amine groups (−NH_2_) on proteins and DNA (Feldman, 1973; Paget and Buttner, 2003), making formaldehyde one of the most potent protein and DNA cross-linking agents.

The presence of genes for the detoxification in *S. epidermidis* strains belonging to B cluster probably allowed them to adapt to formaldehyde resulting either from human and bacterial cells metabolism or from continuous exposure to formaldehyde containing products (dyes, synthetic textiles, disinfectants, and cosmetics). In this regard, *S. epidermidis* might be functioning not only for its own benefit but also in the benefit of the host by avoiding that these compounds reach toxic concentrations to human cells.

### Strains of A/C Cluster Have a Higher Capacity of Survival in Hospital and Infection Environmental Conditions

During infection *S. epidermidis* has to adapt to a new environment, the bloodstream, wherein the pH is stabilized at 7.4, the concentration of salt is steady (0.15 M), specific host matrix proteins are produced, the immune response is exacerbated, and inhibitory concentrations of antibiotics may be present. One of the strategies to deal with such stresses is the attachment to the surface of a device, followed by the development of the biofilm, where bacterial cells are protected.

To evaluate if *S. epidermidis* belonging to A/C and B clusters have developed different capacities to adapt to an infection environment, we compared their ability to bind collagen I, to grow and produce biofilm in blood-similar pH and salt conditions (growth: pH 7.4; 0.15 M NaCl; biofilm: pH 7), to resist oxidative stress and to evade the immune system (by measuring the surface charge and proteolytic activity). Moreover, we searched for specific genes within these lineages that could be associated to a higher infectious capacity.

#### Binding to Host Matrix Proteins and Biofilm Formation at Blood pH

When a medical device is introduced into the human body, its surface becomes coated with a layer of host proteins (Vaudaux et al., 1995; von Eiff et al., 2002; Arrecubieta et al., 2007; Singh et al., 2012). Foreign-body associated infections are attributed to the attachment of bacteria to these host proteins, such as collagen I from tissues, and fibronectin and fibrinogen from blood, through binding of surface proteins (Patti et al., 1994; Foster and Hook, 1998) followed by the production of biofilm. To understand if *S. epidermidis* belonging to the two genetic lineages differed in their ability to cause device-associated infection, we tested their ability to bind collagen I (see Table 3), the collagen type more abundant in human tissues (Singh et al., 2012), rather than the skin. However, our results showed that binding to collagen was not a distinctive factor contributing for their different infectious capacity (OD_*A/C*_ = 0.185 *vs.* OD_*B*_ = 0.175, *p* = 0.067) (see Supplementary Figure S2A). Moreover, we identified two adhesins or microbial surface components recognizing adhesive matrix molecules (MSCRAMMs), namely SdrF and SdrI, that were associated to A/C strains and that have been described to be involved in binding to collagen I and fibronectin (Hartford et al., 2001; Sakinc et al., 2005; Arrecubieta et al., 2007), respectively. In spite of the identified association between SdrF and A/C cluster, this did not impact directly the phenotype of collagen binding, probably due to the existence of known redundancy of proteins other than SdrF that bind collagen I. On the other hand, the comparison of the two lineages regarding the ability to form biofilm at pH 7 (similar to blood’s pH) showed that A/C strains produced significantly more biofilm (OD_*A/C*_ = 1.475; OD_*B*_ = 0.377, *p* = 0.0003) (see Table 3 and Figure 2D) and were simultaneously associated to the presence of the *ica* operon, involved in the production of polysaccharide intercellular adhesion (PIA) (Benjamini–Hochberg *p* = 0.029). These results suggest that upon skin disruption the strains that are able to produce biofilm and that survive better in blood environment are those belonging to A/C cluster that in addition to the ability of binding collagen I have the potential to bind fibronectin (mainly found in the blood after injury) and to produce PIA-dependent biofilms at blood’s pH.

#### Resistance to Host Immune System During Infection

Once in the bloodstream, bacteria are immediately recognized by monocytes and neutrophils through several Pattern recognizing receptors (such as Toll-Like receptors, Nod-Like receptors and C-type lectin receptors), leading to the expression of AMPs, phagocytosis, degranulation, respiratory burst and killing and the formation of neutrophil extracellular traps that capture bacteria inducing a rapid inflammatory response (cytokines and chemokines) (Akira et al., 2006). Bacterial phagocytosis by macrophages occurs via the formation of a membrane-enclosed phagosome containing the microbe in an acidic environment (can be as low as pH 4.5), extremely rich in bactericidal enzymes and toxic compounds (reactive oxygen and nitrogen species and copper) (Weiss and Schaible, 2015).

According to our data, *S. epidermidis* strains have developed several mechanisms that allow them to survive within macrophages. In particular, we observed that although A/C strains had a significantly higher medium growth rate at pH 4.5 (pH of macrophages milieu) (μ_*A/C*_ = 0.335; μ_*B*_ = 0.274, *p* = 0.043) than B strains, they did not vary significantly in their total antioxidant capacity (see Supplementary Figure S2B). The lack of difference in antioxidant capacity might be due to the fact that both A/C and B clusters were associated to genes that provide resistance to oxidative stress (A/C cluster: *mco*; B cluster: *crtOPQMN* operon) and tolerance to copper (A/C cluster: *mco*; B cluster: *cueR*) (see Tables 1, 2). Especially, staphyloxanthin produced by *crtOPQMN* was described in *S. aureus* to participate in the scavenging of free radicals and to have impact in virulence (Clauditz et al., 2006; Mishra et al., 2011).

We have also compared the surface charge and proteolytic activity of the two clusters, as a measure of the capacity to evade the host immune system. According to our data, strains of the A/C and B clusters showed a similar average value of surface charge measured by the relative percentage of unbound cytochrome C, but the values obtained varied from 0 and 78% among strains (see Supplementary Figure S2C and Supplementary Table S5). In particular, closely related strains in the phylogenetic tree (DEN116 and ICE21, 101 SNPs) showed completely distinct relative surface charges (72 and 36%), suggesting that although *S. epidermidis* clusters could not be differentiated based on this feature, modulation of the surface charge appears to be a strategy frequently used by *S. epidermidis* during host-microbe interaction. This ability might confer bacteria with a range of different susceptibilities to human AMPs. Still, *S. epidermidis* clusters appear to differ in their proteolytic capacity when grown on top of skimmed milk agar containing casein (pH = 7), a test utilized to assess production of excreted proteases such as staphylococcal serine proteases (Reed et al., 2001). We found that the proportion of strains that had a high proteolytic capacity was significantly higher in the A/C cluster (48%, *p* = 0.0321) (see Supplementary Figure S2D and Supplementary Table S5) when compared to B cluster (17%). Moreover, we found by pangenomic analysis that a homolog of SplF, an extracellular serine protease-like protein (Reed et al., 2001), was associated to *S. epidermidis* of A/C cluster (77% vs. 35% in B cluster; *p* = 0.008). *SplF* has already been described to be highly immunogenic and to be involved in allergic reactions in *S. aureus* (Zdzalik et al., 2012; Stentzel et al., 2017) and may also interact with the immune system during *S. epidermidis* medical device infections. In *S. aureus* this gene is part of the *spl* operon carried by the pathogenicity island νSaβ (Paharik et al., 2016), however, analysis of the genes in the vicinity of the *splF* homolog in *S. epidermidis* strains from our study showed that this gene was not included within any operon or mobile genetic element.

#### Resistance to Antibiotics

*Staphylococcus epidermidis* colonizing healthy people in the community are usually susceptible to antibiotics (Rolo et al., 2012; Cherifi et al., 2013) but isolates that cause infections within the hospital are typically resistant to multiple antibiotic classes. In particular, around 80% are resistant to β-lactams (Santos Sanches et al., 2000; May et al., 2014). To understand if clusters A/C and B differed in their susceptibility to antibiotics we analyzed their susceptibility profiles to a panel of 14 antibiotics. *S. epidermidis* strains showed resistance to almost all classes of antibiotics tested, varying between 4% and 88% of resistant isolates (see Supplementary Figure S3A) with the exception of linezolid, quinupristin-dalfopristin or vancomycin for which no resistance was observed. Regarding resistance to oxacillin, the frequency and proportion of isolates with high-level resistance (>192 μg/ml) was similar between the two groups (A/C cluster: 53%; B cluster: 50%) (see Supplementary Figure S3B and Table 3). Overall, A/C cluster showed a higher frequency of resistance to penicillin and gentamicin when compared to B cluster (*p*-value < 0.05) (see Table 3). Although in the case of penicillin we could observe a positive association of the *bla*Z and *blaI* genes with the A/C group, no specific genes related to resistance to gentamicin were found associated to this lineage. This was probably due to the fact that diverse resistance mechanisms were detected among isolates of both clusters. In particular, we observed that gentamicin resistant strains belonging to A/C and B groups carried the genes *ant*(4′)-Ib, *aac*(6′)-Ie-*aph*(2′′)-Ia and *aph*(3′)-IIIa. Resistance of A/C strains to gentamicin has probably emerged as a means of survival since gentamicin is frequently used for the treatment of biofilm-associated infections (van de Belt et al., 2001). However, the reason for the increased resistance to penicillin is not so obvious, since this antibiotic is not frequently used to treat device-related infections. A possibility is that *blaZ* regulators are maintained associated to A/C strains because they are important for SCC*mec* acquisition by *S. epidermidis* as was previously described for *S. aureus* (Katayama et al., 2003). Actually, all strains that were resistant to methicillin contained *blaZ*.

### Identification of Epidemiological Markers for *S. epidermidis* A/C and B Clusters

The results from this study highlighted the phenotypic and genotypic differences between the A/C and B clusters and provided several lines of evidence supporting the higher pathogenic potential of A/C strains. Thus, the finding of good epidemiological markers for these two clusters would assist in a better prognosis of the infection and would improve clinical decision-making (namely more appropriate treatment approach) and, ultimately, patient outcomes.

To identify good epidemiological markers for the A/C and B clusters we looked for genes that were associated to the clusters and had frequencies above 90% (see Supplementary Table S3 and Table 2). We found two genes meeting these criteria: a gene encoding a hypothetical protein with a putative DUF1641 domain with a formate dehydrogenase activity associated to cluster B; and one gene encoding for a hydrolase (*yxeP*) of unknown function associated to cluster A/C. In this latter, there were only three isolates belonging to B cluster that contained *yxeP*. These three strains cluster together in the tree, with two strains being more closely related (70 SNPs) than the other (∼2500 SNPs of difference), all collected from nasal colonization in Portugal from draftees sharing the same environment, suggesting that the acquisition of *yxeP* by strains of this lineage might be a rare genetic event.

Our results confirmed the presence of a putative formate dehydrogenase gene (group_862) as highly specific of strains belonging to B cluster, as previously suggested (Conlan et al., 2012). Formate dehydrogenase was described to be important for formate detoxification and NADH/H + production during anaerobic fermentative growth in *E. coli* (Knappe and Sawers, 1990; Sawers, 2005), but in *S. aureus* this enzyme is only expressed in a microaerobic environment (Leibig et al., 2011). It is thus tempting to speculate that *fdh* might enable cluster B strains to survive in specific microaerobic niches in the skin, as hair follicles and sebaceous glands, like *Propionibacterium* spp.

## Conclusion

*Staphylococcus epidermidis* is the main colonizer of the human skin but also one the most important opportunistic pathogens associated to medical device-related infections that occur when the skin barrier is disrupted and the host is immunocompromised.

**FIGURE 3:**
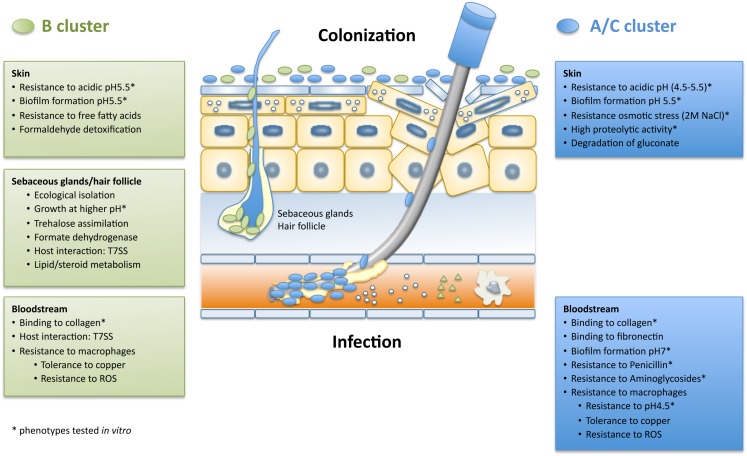
Ecological model of cluster A/C and B strains during a catheter-associated infection. This model was based on the pangenomic wide association studies and phenotypic results performed within this study. In each text box are listed the traits associated to each cluster that are important for survival and adaptation in a specific niche. A/C strains are represented by blue circles and B strains are represented by green circles.

In this study we have compared the two unique clusters that compose *S. epidermidis* population (A/C and B) regarding their genomic content, using genome-wide association studies, and regarding their phenotypic performance in conditions that mimic colonization and infection, which allowed us to create a portrait of each cluster that sheds some light into their ecology and adaptation to commensalism or pathogenicity (see Figure 3). Herein we have shown that strains from cluster A/C and cluster B differ extensively in both their core and accessory genomes and are associated to different genes enrolled in biological functions that were frequently related to their phenotypic performance in the skin and blood conditions, suggesting that the two clusters have been ecologically isolated. Strains from cluster A/C grew faster or reached higher final cell densities in all skin acidic pH (4.5–5.5) and salt concentrations tested (0.15 M, 2 M), whereas B cluster strains were less fit under acidic and osmotic stress. Moreover, B strains were specifically associated to functions related to resistance to free fatty acids, lipid metabolism, microaerobic environment and interspecies interaction (*Propionibacterium* spp.). In infection conditions, however, A/C strains were superior in their ability to resist antibiotics, to grow in macrophage milieu pH conditions (pH 4.5) and to form biofilm at blood pH (∼pH 7). Moreover, they were associated to functions related to binding to host-matrix proteins. Overall, B strains seem to have adapted to survive in a microaerophilic and lipid rich environment such as hair follicles and sebaceous glands; whereas A/C strains probably occupy a more superficial and broader niche in the skin as they were better adapted to the changing osmotic and pH conditions of the skin surface.

Herein we have also produced a population and genome-wide based catalog of genes associated to A/C cluster and identified environmental conditions that favor the growth of A/C strains that could serve as the basis for future studies on *S. epidermidis* pathogenesis and eventually for the development of precision antimicrobial strategies. Additionally, the finding of a genetic marker for the more pathogenic genetic lineage can improve significantly the prognosis and management of medical-device related infections caused by this bacterium.

## Materials and Methods

### Bacterial Isolates

The bacterial collection comprised a total of 83 *S. epidermidis* isolates, collected from different countries (Argentina, *n* = 1; Bulgaria, *n* = 2; Cape Verde, *n* = 4; Colombia, *n* = 1; Denmark, *n* = 14; Greece, *n* = 3; Hungary, *n* = 2; Iceland, *n* = 13; Italy, *n* = 2; Japan, *n* = 2; Mexico, *n* = 5; Portugal, *n* = 31; Taiwan, *n* = 2; and Uruguay, *n* = 1) between 1996 and 2001 and representative of the population found in the hospital (*n* = 60) and community settings (*n* = 23). These isolates were selected to represent all the diversity in sequence types previously found among a temporal and geographically diverse collection of nosocomial and community-associated *S. epidermidis* isolates (Miragaia et al., 2007; Rolo et al., 2012). Isolates were classified as being from colonization (*n* = 45) or infection (*n* = 34) according to defined clinical parameters. Colonization isolates were recovered from several products including nasal swabs (*n* = 35), wounds (*n* = 6), sputum (*n* = 1), urine (*n* = 1) and blood (*n* = 1). Infection products were recovered from blood (*n* = 15), wounds (*n* = 8), urine (*n* = 3), sputum (*n* = 2), catheter (*n* = 2), and cerebrospinal fluid (*n* = 1). Four isolates had no information regarding the clinical significance (infection/colonization) and seven isolates had no information regarding the clinical source.

### Whole-Genome Sequencing

All strains were previously sequenced (Meric et al., 2015) using a HiSeq 2500 machine (Illumina, San Diego, CA, United States) and genome sequences are available on NCBI BioProject PRJNA320931 and BIGSdb^2^ (Jolley and Maiden, 2010). One sample, ICE120 (assembly ID 293_SS_376) although aligning with the reference genome, had a very fragmented assembly and a cumulative length much superior to the expected for this species, suggesting a contamination of the DNA. For this reason, the sample was removed from the dataset and not included in the genomic analysis but still included in the phenotypic analysis.

### Phylogeny Determination

The phylogeny of the strains was reconstructed by generating a Maximum-likelihood (ML) tree with RaxML v8.2.11 (Stamatakis et al., 2012), based on the core genome alignment without the recombining regions identified by Gubbins v2.3.4 (Croucher et al., 2015). Gubbins was run using the default parameters on the complete core genome alignment of the 82 strains. For the first iteration of the program, a Neighbor-joining tree of the same alignment was provided, being previously obtained with FastTree v2.1.10 (Price et al., 2010) using default parameters. The core genome alignment with filtered polymorphic sites was used with RaxML, using the GTRGAMMA substitution model and a bootstrap of 500 simulations. The final tree was visualized using MEGA v7.10.18 (Kumar et al., 2016). The final alignment resulted in 78512 bp polymorphic positions.

### Average Nucleotide Distance

The average nucleotide distance within clusters and between clusters was calculated by averaging the number of single nucleotide polymorphisms (SNPs) of each pair of strains within a cluster or between all strains of each cluster, based on a matrix of distances obtained by Geneious vR8.1.9 based on the core genome alignment, after removal of the recombination regions generated with Gubbins.

### Genomic Variation Assessment

To assess accessory genomic variation a reference pan genome approach was implemented. The 82 genomes were annotated with PROKKA (Seemann, 2014) using a database of eight published complete genomes of *S. epidermidis*: RP62A (Genbank: NC_002976.3); PM221 (Genbank: NZ_HG813242.1); SEI (Genbank: NZ_CP009046.1); 14.1.R1 (Genbank: CP018842.1); 1457 (Genbank: NZ_CP020463.1); ATCC 12228 (Genbank: NC_004461.1), ATCC 12228 (Genbank NZ_CP022247.1) and BPH0662 (Genbank: NZ_LT571449). The annotated genomes were then used in the pan genome creation using Roary v3.11.0 (Page et al., 2015), with locus defined by alleles with a minimum of 85% blastp identity. Scoary v.1.6.16 (Brynildsrud et al., 2016) was used to find significant associations between the genetic loci and the different phylogenetic clusters. If several genes within each cluster were attributed the same functional annotation, a unique gene was considered for cluster comparison, as most probably this correspond to different alleles of the same gene that fell below the 85% cut-off. The only exceptions were paralogs that were considered as different genes although having the same annotation. Only loci with a Benjamini–Hochberg *p* < 0.05 and an odds ratio (OR) > 1 were considered to be positively associated to a cluster. Additionally, they were only considered for analysis if they had no matching function in the genes positively associated to the other cluster.

### Antimicrobial Susceptibility Testing

All bacterial isolates were tested for antimicrobial susceptibility trough disk diffusion method (Kirby-Bauer), according to the Clinical and Laboratory Standards Institute (CLSI) guidelines of 2014 (CLSI, 2014). Susceptibility was tested to a panel of 12 antibiotics: penicillin, erythromycin, clindamycin, linezolid, ciprofloxacin, quinupristin-dalfopristin, sulfamethoxazole-trimethoprim, tetracycline, fusidic acid, rifampicin, chloramphenicol, and gentamicin. In the case of fusidic acid, the European Committee on Antimicrobial Susceptibility Testing (EUCAST) guidelines from 2016 were used. Strains were considered as multidrug-resistant (MDR) if they were non-susceptible to at least three or more antimicrobial classes (Magiorakos et al., 2012).

The minimum inhibitory concentration (MIC) for oxacillin and vancomycin was determined by e-test (BioMérieux, Marcy-l’Étoile, France). Bacterial suspensions (0.5 McFarland) were inoculated in Mueller-Hinton agar (MHA, BBL^TM^, Becton Dickinson, Sparks, MD, United States), which in the case of oxacillin was supplemented with 2% NaCl, and incubated at 37°C for 48 h. Strains were considered to be susceptible to oxacillin if the MIC ≤ 0.25 μg/ml, resistant if the MIC ≥ 0.5 μg/ml; and were considered to be susceptible to vancomycin if the MIC ≤ 4 μg/ml, resistant if the MIC ≥ 32 μg/ml, according to 2014 CLSI guidelines (CLSI, 2014).

### Growth Assays

Growth curves at pH 4.5, 5.5, 7.0, and 7.4 were defined for all isolates. All growth assays were performed using a microtiter reader (Infinite^®^ 200 PRO series, Tecan Group Ltd., Männedorf, Switzerland) in 96-well microtiter plates. Briefly, overnight cultures were inoculated to an initial OD_600 *nm*_ of 0.05 in tryptic soy broth (TSB, Bacto^TM^, BBL, Becton Dickinson, Sparks, MD, United States) and grown with aeration (180 rpm) at 37°C for 30 h. Each strain was tested in triplicate and each experiment was repeated three times independently for different growth conditions. The pH of the medium was adjusted with HCl (37%) and the salt concentration was adjusted with NaCl (Merck KGaA, Darmstadt, Germany).

### Biofilm Assays

The biofilm formation was tested at pH 5.5 and pH 7 and assays were performed on 96-well microtiter plates (Corning^®^ 96 Well TC-Treated flat bottom, Sigma-Aldrich, St. Louis, MO, United States). Overnight cultures were inoculated to an initial OD_600 *nm*_ of 0.05 in TSB and grown in static conditions with no aeration at 37°C for 24 h. After incubation, the contents of the wells were washed with water, heat fixed at 60°C, stained with 0.06% crystal violet and resuspended in acetic acid (30%). The optical density was measured at 595 nm in a microtiter plate reader (Infinite^®^ 200 PRO series, Tecan Group Ltd., Männedorf, Switzerland). Each strain was tested in triplicate and each experiment was repeated three times independently. *S. epidermidis* strains RP62A and ATCC12228 were used as positive and negative controls of biofilm formation (Christensen et al., 1995), respectively.

### Collagen Assays

*Staphylococcus epidermidis* adhesion to collagen was assayed by adapting the protocol described by Bowden et al. (2002). Briefly, early log-phase *S. epidermidis* cultures (OD_600_ between 0.3 and 0.7) were harvested and centrifuged (5000 *g* for 5 min). Cells were washed, resuspended in PBS, adjusted to a final OD_600_ of 1 and inoculated in 96-well microtiter plates coated with collagen I (Corning^TM^ BioCoat^TM^ Collagen I 96-well Clear Flat Bottom TC-treated Microplate, VWR, Radnor, PA, United States) for 2 h at room temperature (RT). After gentle washes with PBS, adherent cells were fixed with 25% (v/v) aqueous formaldehyde and incubated at RT for 30 min. The plates were washed gently with PBS, stained with 0.5% crystal violet for 5 min, washed again and read on a microtiter plate reader (Infinite^®^ 200 PRO series, Tecan Group Ltd., Männedorf, Switzerland) at 595 nm.

### Antioxidant Capacity Assay

Overnight cultures were adjusted to 0.1 (OD_650_) in TSB and let grow to 0.7 (OD_650_), incubated with or without H_2_O_2_ (30%, VWR, Radnor, PA, United States) for 20 min and harvested by pelleting. Cells were washed twice, resuspended in with 1x PBS and lysed with 2.5 μl lysozyme (20 mg/ml, Sigma-Aldrich, St. Louis, MO, United States), 5 μL lysostaphin (10 mg/ml, Ambi products LLC, Lawrence, NY, United States), and 3 μl RNAase (10 mg/ml, Sigma-Aldrich, St. Louis, MO, United States) for 2 h and the lysate was harvested after centrifugation at 4°C and 5000 *g* for 10 min. Antioxidant capacity of the samples was tested using the Total Antioxidant Capacity Assay kit (Sigma-Aldrich, St. Louis, MO, United States), following the manufacturer’s instructions.

### Surface Charge Assay

The protocol used to assay the surface charge was adapted from Peschel et al. (1999). Bacterial cultures were grown overnight and harvested by centrifugation (5 min, 5000 *g*). Cells were washed twice with MOPS buffer (20 mM, pH 7) and resuspended in the same buffer to 15 (OD_600_). Cell suspensions and cytochrome c (1 mg/ml) were mixed in equal volumes and incubated for 10 min at RT (protected from light and with continuous soft agitation). Cells were centrifuged (13,000 rpm, 3 min) and unbound cytochrome c was measured by absorbance (OD_410_). The higher the absorbance value, the more positively charged is the cell envelope.

### Proteolysis Assay

Overnight cultures were grown to 0.7 (OD_600_) in TSB and were spotted onto 10% skimmed milk agar (Sigma-Aldrich, St. Louis, MO, United States). Plates were incubated at 37°C for 24–48 h and lysis halos were measured from the border of the spotted bacterial growth until the border of the halo. The strains were categorized into three percentiles (high, medium, and low) depending on the size of the halos produced.

### Phenotypic Assays Statistical Analysis

The statistical analysis was performed using the software GraphPad Prism 6. The significance of the differences between cluster A/C and cluster B regarding growth rates, biofilm production, collagen adhesion, cell wall net charge and antioxidant capacity was assessed using the two-tailed non-parametric Student’s *t*-test. The significance of difference regarding proteolysis and antimicrobial resistance was analyzed using Chi-square test. A *p*-value < 0.05 was considered statistically significant.

## Data Availability

Genome sequences used in this study are available on NCBI BioProject PRJNA320391 and BIGSdb (https://www.sheppardlab.com/resources/).

## Ethics Statement

Isolates from colonization in the hospital in Portugal were all obtained from nasal screening of patients from Hospital da Força Aérea (Lisbon, Portugal). Collection of these isolates was performed with approval from the local medical Ethics Committee (“Comissão de Ética para a Sáude da Força Aérea,” Hospital da Força Aérea, Lisboa, Portugal) and Infection Control Committee ("Comissão de Higiene Hospitalar do Hospital da Força Aérea," Hospital da Força Aérea, Lisboa, Portugal) and written informed consent was not required, according to Ethics Committee-approved guidelines. Isolates from colonization in the community were obtained from screening of draftees attending Centro de Formação da Ota (Lisbon, Portugal), which was performed with written informed consent and approval from all the necessary military authorities. Nosocomial strains from other countries were collected as part of the hospital routine diagnostic testing. The strains were de-identified and analyzed anonymously and the strains, not a human, were studied. Ethical approval and informed consent were thus not required.

## Author Contributions

DE cultured the isolates and performed the phenotypic experiments. DE and MM carried out the data analysis and interpretation, and wrote the manuscript. CM and JC were involved in the data analysis and manuscript revision. GM and SS performed the sequencing of the isolates and reviewed the manuscript. RS and HL helped in the writing and revision of the manuscript. All authors read and approved the final manuscript.

## Conflict of Interest Statement

The authors declare that the research was conducted in the absence of any commercial or financial relationships that could be construed as a potential conflict of interest.

## Footnotes

1 www.hmdb.ca

2 https://www.sheppardlab.com/resources/

## Supplementary Material

FIGURE S1 R-plot depicting the open pan genome of *S. epidermidis*.

FIGURE S2 Performance of strains from cluster A/C and B in the collagen binding assay **(A)**, antioxidant capacity assay **(B)**, surface charge assay **(C)**, and proteolytic capacity assay **(D)**.

FIGURE S3 Antimicrobial susceptibility testing of A/C and B cluster strains. **(A)** Antimicrobial susceptibility profile of cluster A/C and B strains by disk diffusion assay; **(B)** Minimum inhibitory concentration for oxacillin of cluster A/C and B MRSE strains by *e*-test. Legend: penicillin (PEN), oxacillin (OXA), erythromycin (ERY), clindamicin (DA), cloramphenicol (C), rifampicin (RIF), linezolid (LZD), ciprofloxacin (CIP), quinopristin-dalfopristin (QD), trimethoprim-sulphamethoxazole (SXT), tetracyclin (TET), fusidic acid (FD), gentamicin (GEN), and vancomycin (VAN).

TABLE S1 Nucleotide distance matrix generated in Geneious based on the core genome alignment without the recombination regions.

TABLE S2 List of genes composing the pan genome generated by Roary.

TABLE S3 List of genes positively associated to cluster A/C (Benjamini–Hochberg *p* < 0.05 and OR > 1).

TABLE S4 List of genes positively associated to cluster B (Benjamini–Hochberg *p* < 0.05 and OR > 1).

TABLE S5 Summary of the epidemiological and phenotypic characteristics of cluster A/C and cluster B strains.
