# Supplementary figures and images for: Distinct Phenotypic and Genomic Signatures Underlie Contrasting Pathogenic Potential of Staphylococcus epidermidis Clonal Lineages
Source: Front Microbiol. 2019 Aug 27;10:1971. doi: 10.3389/fmicb.2019.01971 (PMC6719527; doi:10.3389/fmicb.2019.01971)

# No. of genes in the pan-genome

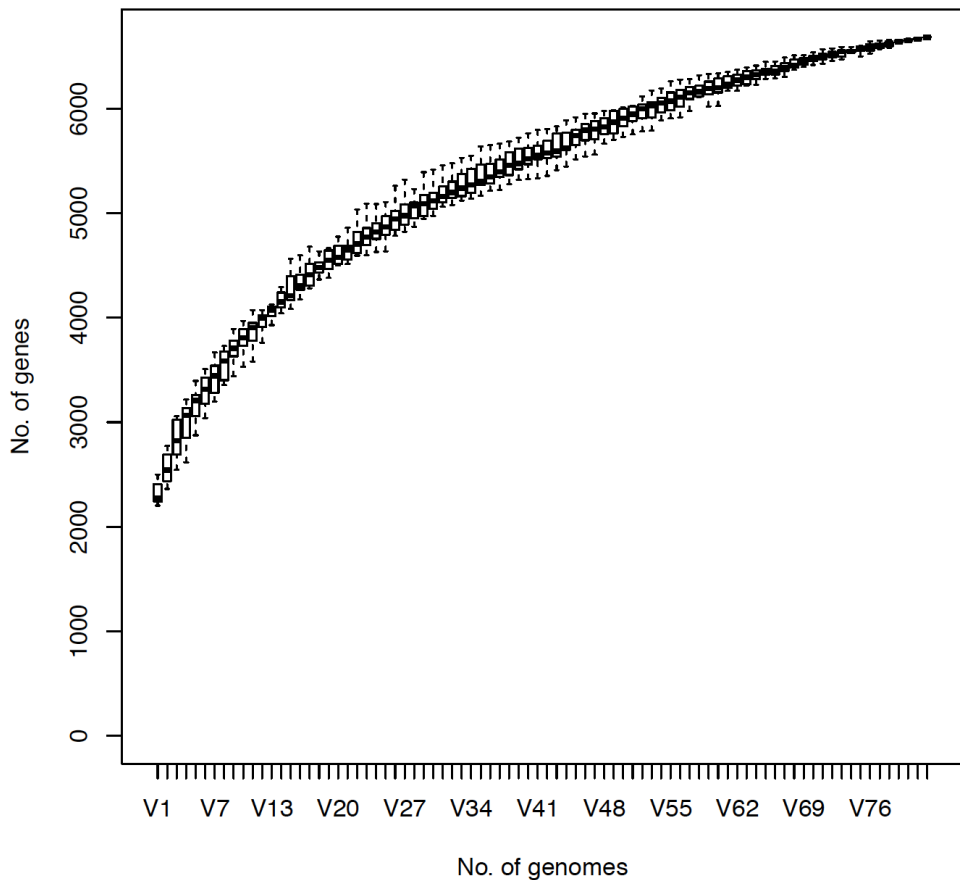

Supplement: FIGURE S1 — R-plot depicting the open pan genome of S. epidermidis. [file Data_Sheet_1.PDF]

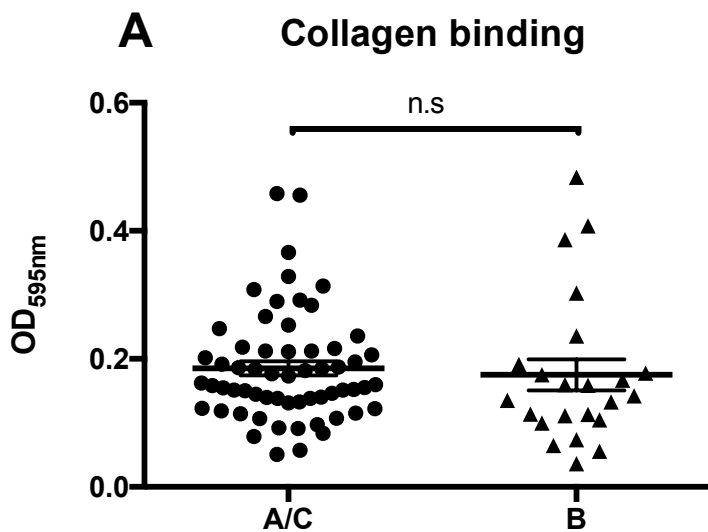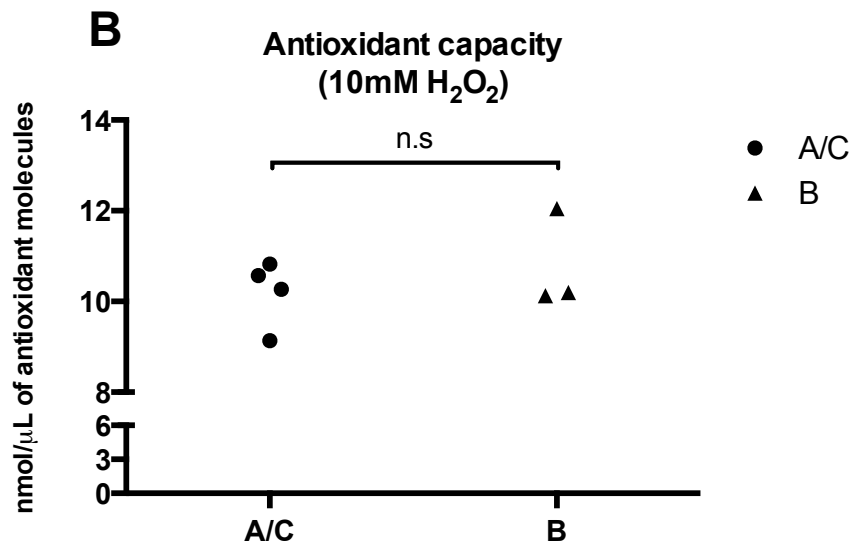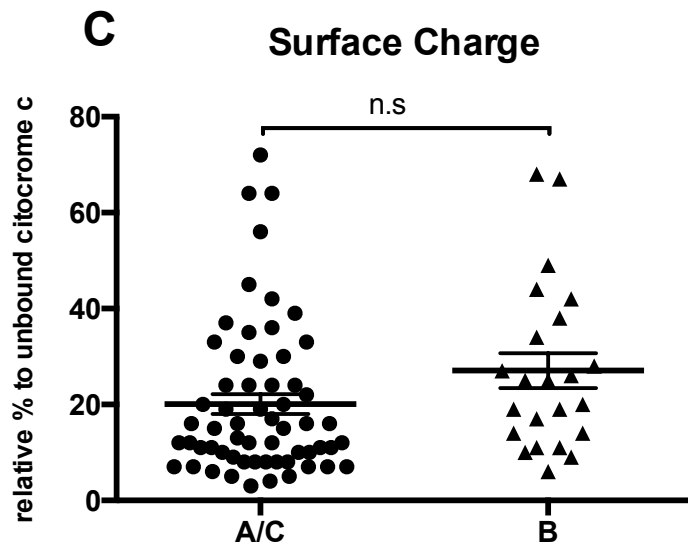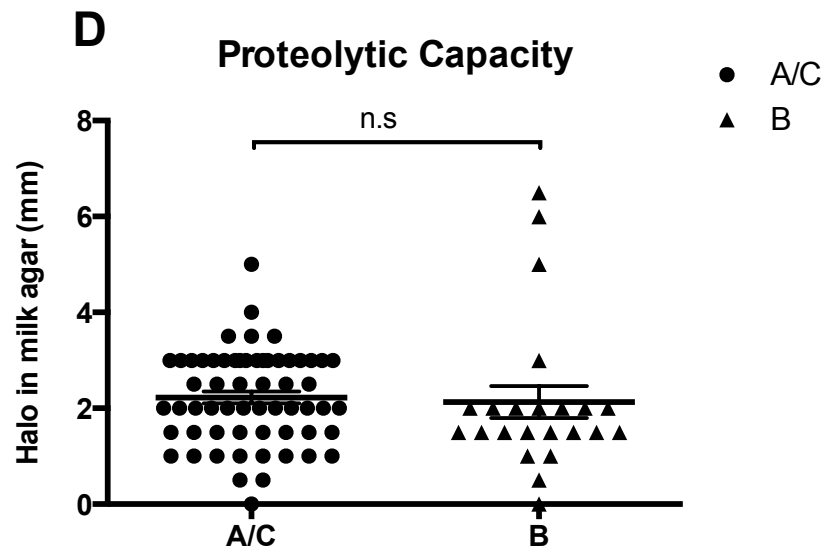

Supplement: FIGURE S2 — Performance of strains from cluster A/C and B in the collagen binding assay (A), antioxidant capacity assay (B), surface charge assay (C), and proteolytic capacity assay (D). [file Data_Sheet_2.PDF]

## Antimicrobial resistance

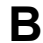

# MRSE

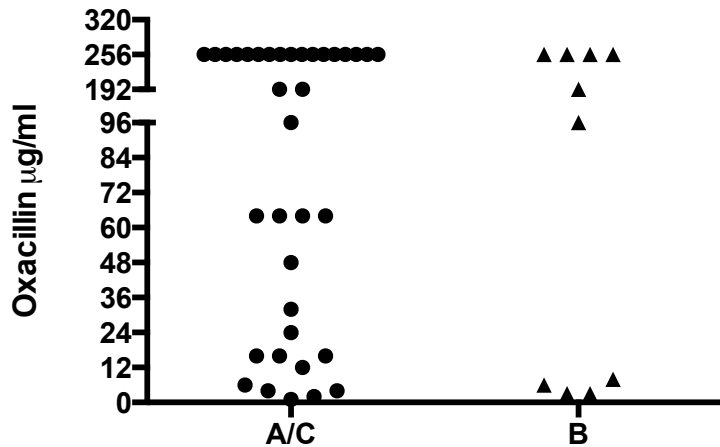

Supplement: FIGURE S3 — Antimicrobial susceptibility testing of A/C and B cluster strains. (A) Antimicrobial susceptibility profile of cluster A/C and B strains by disk diffusion assay; (B) Minimum inhibitory concentration for oxacillin of cluster A/C and B MRSE strains by e-test. Legend: penicillin (PEN), oxacillin (OXA), erythromycin (ERY), clindamicin (DA), cloramphenicol (C), rifampicin (RIF), linezolid (LZD), ciprofloxacin (CIP), quinopristin-dalfopristin (QD), trimethoprim-sulphamethoxazole (SXT), tetracyclin (TET), fusidic acid (FD), gentamicin (GEN), and vancomycin (VAN). [file Data_Sheet_3.PDF]
